# Microbiological Safety and Quality of Fermented Products

**DOI:** 10.3390/foods12112204

**Published:** 2023-05-31

**Authors:** Xiaoyan Liu, Jinghao Ma, Guangsen Fan

**Affiliations:** Beijing Engineering and Technology Research Center of Food Additives, School of Food and Health, Beijing Technology and Business University, Beijing 100048, China; liuxiaoyan@btbu.edu.cn (X.L.); 15032049018@163.com (J.M.)

Fermented foods, which have emerged fortuitously over the course of human development, have become an essential part of human history worldwide. These rich and diverse fermented foods not only have unique flavors and qualities that appeal to local preferences but also embody local cultures and play a significant role in human life. Despite the popularity of fermented foods globally, many traditional fermented foods are still produced using open fermentation methods where it is difficult to evaluate the safety or function of microorganisms, leading to inevitable challenges. Therefore, to advance the knowledge on the functional properties of microorganisms in fermented foods globally, and shed light on the impact of these microorganisms on the safety and quality of fermented foods, we present “Microbiological Safety and Quality of Fermented Products”, a Special Issue publishing 10 papers.

This Special Issue covers four pivotal research topics. First, the microbial safety of fermented products, where researchers have focused on identifying food-borne pathogens in particular fermented products through detection methods, thereby improving the safety levels and reducing the harms of fermented products. Second, the functions of microorganisms in fermented foods have been investigated. Third, researchers have explored the application of microorganisms in various stages of fermented food production, for example, in pre-treatment, fermentation, and post-fermentation processes. Fourth, researchers have analyzed the changes in microflora during the different stages of fermenting food, providing crucial evidence for understanding the role played by microbes in creating distinctive flavor and quality.

The first topic of the Special Issue, “Microbiological Safety and Quality of Fermented Products,” includes two papers [1,2] that are both focused on the microbiological safety of fermented products. The first paper by Stefanou et al. [1] investigated the microbiological safety, quality, and physicochemical composition of small-scale farmer’s fresh cheeses made from cow’s and goat’s milk. The results showed that none of the samples contained *Listeria monocytogenes* or *Salmonella* spp. However, coliforms were present in all of the goat’s milk cheeses and only in two of the cow’s milk cheeses. Further, low levels of cadmium, below 0.008 ppm, were detected in three cow’s milk samples. The authors concluded that the raw milk cheeses studied are considered safe and free of the targeted pathogens, while also possessing high nutritional value. This research provided crucial information on the safety and quality of fresh cheeses produced on a small scale. Such studies are essential to ensure that food safety standards are met, and they help to address concerns associated with the consumption of raw milk cheeses. Future studies could focus on more extensive sampling methods or other preventative measures to reduce the risk of bacterial contamination in artisanal cheese making.

The second paper related to this topic, conducted by Srimahaeak et al. [2], aimed to assess the potential for spoilage by various yeast strains (*Kluyveromyces marxianus* (Km1, Km2, and Km3), *Pichia kudriavzevii* Pk1, and *Torulaspora delbrueckii* Td1) when grown in skyr stored under cold conditions. The yeast strains were isolated from the skyr samples using sequencing of the 26S rRNA gene. *K. marxianus* yeasts exhibited substantial growth rates in skyr, producing a notable amount of volatile organic compounds (VOCs) responsible for off-flavors, such as alcohols (3-methyl-1-butanol, 2-methyl-1-propanol, and 1-hexanol), esters (ethyl acetate and 3-methylbutyl acetate), and aldehydes (hexanal, methylbutanal, and methylpropanal). In contrast, the growth of *P. kudriavzevii* Pk1 led to moderate increases in several alcohols and esters (primarily, 3-methyl-1-butanol and ethyl acetate). *T. delbrueckii* Td1 demonstrated no significant changes in VOC levels. Further, all *K. marxianus* strains and *P. kudriavzevii* Pk1 significantly reduced key aroma compounds, diacetyl, and acetoin. Additionally, unlike other yeast species studied, *K. marxianus* was able to use lactose, leading to the production of ethanol and carbon dioxide. Overall, *K. marxianus* was found to have the highest potential for spoilage activity. The researchers highlighted the differences in fermentative and spoilage activities between the various yeast species, and, through these findings, clarified the roles of yeast metabolites in off-flavor formation and quality defects in skyr during cold storage. This research provides insights into the spoilage potential of various yeast strains in skyr under cold storage conditions. Further studies could focus on identifying possible methods to mitigate these effects, or on developing alternative starter cultures less prone to spoiling for use in skyr production.

This Special Issue contains three manuscripts that delve into the second topic which is related to the function of microorganisms in fermented foods [3,4,5]. The first study, conducted by Cheng et al. [3], employed an RNA-seq technique to identify differentially expressed genes (DEGs) in a UVA/H2O2-induced model. Gene ontology clustering and Kyoto Encyclopedia of Genes and Genomes pathway analysis were employed to determine core DEGs and key signaling pathways. The PI3K-AKT signaling pathway was identified as playing a role in the oxidative process and was further validated through reverse transcription-quantitative polymerase chain reaction (RT-qPCR). Using three types of *Schizophyllum commune* fermented actives, the authors evaluated whether the PI3K-AKT signaling pathway plays a role in mediating the resistance of active substances to oxidative damage. The results indicated that the DEGs were mainly enriched in five categories: external stimulus response, oxidative stress, immunity, inflammation, and skin barrier regulation. *S. commune* ferments effectively reduced cellular oxidative damage via the PI3K-AKT pathway at both the cellular and molecular levels. Several mRNAs (COL1A1, COL1A2, COL4A5, FN1, IGF2, NR4A1, and PIK3R1) were detected, and the results were consistent with those obtained from RNA-seq analysis. The authors suggested that these findings may provide a common set of standards or criteria for screening anti-oxidative actives in the future. This research highlights the importance of microorganisms in fermented foods and their potential to mediate various biological processes, for example, reducing oxidative damage. These findings represent a significant advancement toward understanding the mechanisms underlying the beneficial effects of microbial fermentation on human health. Further investigations could focus on the optimization of fermentation conditions and the identification of novel microbial strains with enhanced beneficial properties.

In the second study, conducted by Fu et al. [4], the potential obesity-preventing function of *Aloe Vera*-Fermented Beverage (AFB) was investigated in HepG2 cells subjected to a high-fat environment and in high-fat diet (HFD)-fed mice. The results demonstrated that AFB intervention decreased lipid droplet accumulation in HepG2 cells, suppressed body weight gain and adipose accumulation, and reduced the serum levels of total cholesterol, alanine aminotransferase, and interleukin 10 in HFD-fed mice. Furthermore, AFB treatment altered the composition of the gut microbiota in these mice. Specifically, the ratio of Firmicutes/Bacteroidetes was reduced, while the relative abundance of *Muribaculaceae*, *Alistipes* and *Rikenellaceae*_RC9_gut_group was increased upon AFB administration compared with HFD-fed control mice. Taken together, these findings suggest that AFB may effectively prevent diet-induced obesity and serve as a promising option for modulating obesity-related gut dysbiosis. It contributes to our understanding of the beneficial effects of AFB in preventing and treating obesity, which remains an ongoing challenge in public health. Further investigations could focus on elucidating its underlying mechanisms and optimizing the dosage and duration of the AFB intervention. These findings provide important insights into the development of novel strategies for addressing the growing epidemic of obesity.

The third study conducted by Du et al. [5] aimed to compare the tolerance to lactic acid between *Pichia kudriavzevii* C-16 and its type strain ATCC 24210 in a controlled experiment. The findings revealed that *P. kudriavzevii* C-16 exhibited higher biomass yields and lactic acid consumption rates under lactic acid stress conditions. To further investigate the response of *P. kudriavzevii* C-16 to lactic acid, mRNA sequencing was employed as a means of analysis. The results demonstrated that after 12 h and 24 h of cultivation, 92 and 96 genes, respectively, exhibited significant upregulation, while 52 and 58 genes, respectively, displayed significant downregulation in *P. kudriavzevii* C-16. Notably, these genes were found to be involved in various pathways, including the pyruvate metabolic pathway, ABC transporter proteins, glutamate metabolic pathway, as well as the biosynthetic pathway of leucine and valine. Differential expressions of these genes were observed when comparing *P. kudriavzevii* C-16 and its type strain ATCC 24210. Furthermore, the investigation of higher alcohol production revealed a notable increase in the concentrations of isobutyl alcohol and isoamyl alcohol produced by *P. kudriavzevii* C-16. This finding corresponded with the upregulation of genes associated with the biosynthesis of relevant amino acids.

The Special Issue focuses on the application of microorganisms in fermented foods, and it comprises four original papers [6,7,8,9] that address the third topic. In the first study [6], conducted by Feng et al., the production of baijiu using four strains of *actinomycetes* (*Streptomyces violascens* SPQ1, *S. sampsonii* SPS1, *S. thermophilus* SPG1, and *S. griseus* SPH1) was investigated. Both solid-state and liquid fermentation techniques were employed, with five different brewing raw materials serving as substrates. To analyze and compare the terpenoid content in the metabolites, gas chromatography–mass spectrometry was utilized. During liquid fermentation, the four actinomycete strains produced a total of 31 terpenoids from the hydrolysates of the five fermentation substrates. The cumulative terpenoid content in the fermentation products was measured as 989.94 μg/kg. On the other hand, after 28 days of solid-state fermentation, the same actinomycete strains produced a total of 64 terpenoids using the five fermentation substrates, and the cumulative terpenoid content was determined to be 23,651.52 μg/kg in the fermentation products. Based on these results, the author concluded that both the choice of fermentation substrates and the fermentation methods employed exert a significant influence on the production of terpenoids by actinomycetes.

The second study conducted by Lin et al. [7] sheds light on the impact of pre-fermentation, using different yeast strains, on bacterial cellulose (BC) production in coconut water media. The findings emphasize the variability in BC yield when utilizing pre-fermented coconut water compared to fresh coconut water, with the pre-fermentation by *Saccharomyces cerevisiae* SC7 resulting in a remarkable 165% increase in BC yield. Moreover, the research highlights the influence of natural pre-fermentation and SC7 pre-fermentation on the amino acid composition of coconut water, and the subsequent effects on BC production. The investigation further reveals the varied effects of selected amino acids on BC production, with methionine at a concentration of 3.0% (*w*/*v*) yielding the highest BC output. Importantly, the addition of 3.0% methionine not only enhanced BC yield but also influenced its physical properties, leading to the formation of larger loops of loosely aggregated microfibers, an increase in BC crystallinity, and alterations in thermal and mechanical characteristics. These findings contribute to our understanding of the intricate relationship between microorganisms, fermentation processes, and the production of value-added products such as BC. The results underscore the potential for optimizing fermentation conditions and exploring novel microbial strains to enhance BC production and tailor its properties for specific applications. Further investigations in this field could focus on fine-tuning fermentation parameters, elucidating the underlying mechanisms of amino acid-mediated BC synthesis, and identifying microbial strains with enhanced functionalities and broader industrial potential.

The third study conducted by Ma et al. [8] focused on the isolation and characterization of strain YHM-G from the baijiu-producing environment, which exhibited a remarkable capacity for 3-methylthiol (3-Met) production. Through comprehensive analysis, strain YHM-G was identified as *Hyphopichia burtonii* based on its morphological properties, physiological and biochemical characteristics, and genetic sequencing of the ribosomal large subunit 26S rRNA gene D1/D2 domain. The study further optimized the conditions for 3-Met production by strain YHM-G using a series of experimental designs. The optimized conditions included a glucose concentration of 42.7 g/L, pH 6, yeast extract concentration of 0.9 g/L, L-methionine (L-Met) concentration of 6 g/L, culture temperature of 28 °C, shaking speed of 210 rpm, loading volume of 50 mL/250 mL, inoculum size of 0.5% (*v*/*v*), culturing period of 48 h, and the addition of 2.5 g/L of Tween-80. Under these optimized conditions, the production of 3-Met by strain YHM-G reached 3.16 g/L, representing an 88.1% increase compared to the initial levels before optimization. Additionally, strain YHM-G demonstrated an ability to produce a diverse range of flavor compounds that are significant for various food products. This suggests that the strain has the potential to enhance the abundance of 3-Met in fermented foods, thereby improving their aroma profiles. Overall, the findings of this study highlight the potential application of strain YHM-G in the food industry, as it offers the opportunity to enhance the flavor and sensory attributes of fermented foods through increased 3-Met production. Further research could explore the commercial-scale production of 3-Met using strain YHM-G and investigate its impact on the sensory quality of specific food products.

The fourth study conducted by Liu et al. [9] aimed to identify the aroma profile of sun-dried black tea (SBT) using advanced analytical techniques. Through the application of headspace solid-phase microextraction coupled with gas chromatography–mass spectrometry and gas chromatography-olfactometry, a comprehensive analysis of the aroma compounds present in SBT was performed. The results of the study revealed the capture of 37 scents using the GC-O technique, with odor intensities ranging from 1.09 ± 1.93 to 9.91 ± 0.29. Among these scents, a total of 35 compounds were successfully identified. Furthermore, 21 compounds were identified as key odor-active compounds, exhibiting odor activity values greater than or equal to 1. These key odor-active compounds were further analyzed, and their concentrations were determined, allowing for the successful imitation of the aroma profile of the selected SBT sample to a certain extent. To confirm the importance of specific compounds in the aroma profile of SBT, an omission test was conducted using 25 models. The results of the test highlighted several compounds as being key odor-active compounds for the aroma profile of SBT, namely (E)-β-damascenone, β-ionone, dihydro-β-ionone, linalool, and geraniol. Additionally, phenylethyl alcohol, (E)-2-decenal, hexanal, and methyl salicylate were also found to contribute significantly to the aroma profile of SBT. This study provides valuable theoretical support for enhancing the aroma quality of sun-dried black tea. The identification and understanding of key odor-active compounds offer insights for the improvement and optimization of SBT production processes, with the aim of enhancing its aroma characteristics. Further research could focus on exploring strategies to manipulate the concentrations of these key compounds to meet consumer preferences and promote the development of high-quality sun-dried black tea products.

Sun et al. [10] conducted a study focusing on the fungal community present in Suanyu, a traditional fermented fish food product from southwest China, and its correlation with the food’s physicochemical properties. The authors employed high-throughput sequencing to analyze the fungal community structure of Suanyu samples from the provinces of Guizhou and Hunan. Furthermore, Spearman’s correlation coefficient was used to assess the relationship between dominant fungi and the physicochemical characteristics of Suanyu. The results revealed ranges for the pH value (4.30–5.50), total volatile base nitrogen content (17.11–94.70 mg/100 g), and thiobarbituric acid reactive substance content (0.61–3.62 mg/kg) in the Suanyu samples. Additionally, the average contents of total volatile base nitrogen, thiobarbituric acid reactive substance, and total biogenic amines in Suanyu from Guizhou were found to be lower compared to those from Hunan. The main biogenic amines detected in Suanyu were phenethylamine, putrescine, cadaverine, histamine, and tyramine. The dominant fungal phylum observed was *Ascomycota*, with *Kodamaea*, *Debaryomyces*, *Wallemia*, *Zygosaccharomyces*, and unclassified *Dipodascaceae* identified as the dominant fungal genera in different Suanyu samples. Furthermore, Suanyu samples from Guizhou exhibited high abundance levels of *Kodamaea* and *Zygosaccharomyces*. The correlation analysis revealed that *Kodamaea* and *Zygosaccharomyces* exhibited negative correlations with thiobarbituric acid reactive substance (TBARS) and total volatile base nitrogen (TVBN) content. Unclassified *Dipodascaceae* was also found to have a significant negative correlation with tyramine. These findings contribute to a deeper understanding of the fungal community and the fermentation characteristics of dominant fungi in Suanyu. The identification of dominant fungi, and their correlations with specific compounds, provides valuable insights into the fermentation process and quality control of Suanyu. Further investigations can build upon these findings to explore the functional roles of specific fungal species and develop strategies for optimizing the production and quality of Suanyu.

In summary, the collection of ten papers published in this Special Issue serves as a comprehensive and significant contribution to the topic of “Microbiological Safety and Quality of Fermented Products”. These papers showcase the extensive research efforts dedicated to this field and emphasize the necessity for further investigations. We express our gratitude to all the authors for their valuable contributions, which have greatly enriched this Special Issue. We believe that the findings presented in these papers will captivate and enlighten readers, fostering a deeper understanding of the subject matter.
